# Genome Sequence of the Wine Yeast *Saccharomycodes ludwigii* UTAD17

**DOI:** 10.1128/MRA.01195-18

**Published:** 2018-11-08

**Authors:** Maria J. Tavares, Ulrich Güldener, Marcos Esteves, Arlete Mendes-Faia, Ana Mendes-Ferreira, Nuno P. Mira

**Affiliations:** a Department of Bioengineering, Instituto Superior Técnico, Universidade de Lisboa, Lisbon, Portugal; b iBB–Institute for Bioengineering and Biosciences, Biological Sciences Research Group, Instituto Superior Técnico, Lisbon, Portugal; c Department of Bioinformatics, Wissenschaftszentrum Weihenstephan, Technische Universität München, Freising, Germany; d Escola de Ciências da Vida e Ambiente, Universidade de Trás-os-Montes e Alto Douro, Vila Real, Portugal; e BioISI-Biosystems and Integrative Sciences Institute, Lisbon, Portugal; Broad Institute of MIT and Harvard

## Abstract

This work describes, for the first time, the genome sequence of a Saccharomycodes ludwigii strain. Although usually seen as a wine spoilage yeast, S. ludwigii has been of interest for the production of fermented beverages because it harbors several interesting properties, including the production of beneficial aroma compounds.

## ANNOUNCEMENT

Saccharomycodes ludwigii is one of the many non-*Saccharomyces* (NSY) species present in the wine must mycobiome (1–3). Phylogenetically, the *Saccharomycodes* genus is placed in the *Saccharomycodaceae* family, in the phylum *Ascomycota* and subphylum *Saccharomycotina*, being considered a sister genus to *Hanseniaspora* (4). Unlike the genus *Hanseniaspora*, which is seen as having a positive effect in vinification (5), the presence of S. ludwigii is of concern since it is considered a spoilage agent, reducing the organoleptic properties of wine and interfering with the clarification process (4, 6). Despite this negative effect of S. ludwigii in wine production, this species has been explored for the production of other fermented beverages (7–10), and it has been reported to be an interesting flavoring agent that is able to produce several relevant aroma compounds (9).

Little is known concerning the genetics and physiology of the S. ludwigii species. In particular, no genomic sequence has yet been reported for a strain of this species, which impedes our better understanding and exploration of it. As such, in this work, we have obtained the genomic sequence of an S. ludwigii isolate (UTAD17) recovered from wine must in the Douro region of Portugal by using a selective medium for NSY species. To obtain the genome sequence of S. ludwigii UTAD17, we cultivated cells in rich medium and extracted the DNA as described previously (11). The DNA libraries were prepared using the ThruPLEX DNA-seq kit, and paired-end sequencing of the generated DNA fragments was performed on a MiSeq platform. After two sequencing rounds, 20,333,547 reads of 250 bp on average were obtained and *de novo* assembled into 1,360 contigs (*N*_50_ length of 17,540 bp; filtered to have a coverage above 300× and a size above 1,000 nucleotides). The sum of the assembled contigs totaled 10,785,241 bp, which is in line with the genome size predicted for Hanseniaspora osmophila (4, 12), another species in the *Saccharomycodaceae* family. Automatic annotation of the S. ludwigii UTAD17 genomic sequence was undertaken using (i) Fgenesh trained on Aspergillus nidulans, Neurospora crassa, and a mixed matrix based on different species (13); (ii) GeneMark-ES (14); and (iii) Augustus (15). The different gene models proposed by the algorithms were displayed in the Generic Genome Browser (GBrowse) (16), allowing individual manual validation. Gene models showing the highest similarity with homologues described in other yeast species were selected. If needed, gene structures were adjusted by splitting or fusing the gene models or redefining exon-intron boundaries. The predicted complete set of open reading frames (ORFeome) of S. ludwigii UTAD17 is estimated to be 4,015 protein-coding genes. BLASTP analysis (using the nonredundant protein sequences database as a reference) (17) revealed that S. ludwigii proteins share a high degree of homology with H. osmophila and Lachancea fermentati. This is an interesting observation since both species are indigenous to wine musts and influence the wine fermentation process (12, 18, 19). It is expected that the S. ludwigii UTAD17 genome sequence reported here can foster research focused on this species, contributing particularly to overcoming its activity as a wine spoilage agent and improving its utilization in the production of fermented beverages.

### Data availability.

The reads and the assembled genome sequences of Saccharomycodes ludwigii UTAD17 have been deposited in ENA under accession number UFAJ01000000 (contigs UFAJ01000001 through UFAJ01001360; study accession number PRJEB27462; read accession number SAMEA4945973).
